# Multiple sclerosis-associated uveitis successfully controlled with ofatumumab

**DOI:** 10.1186/s12348-025-00543-0

**Published:** 2025-11-14

**Authors:** Soufiane Azargui, Julia L. Xia, Ian J. McClain, Mark Dacey, Andrew B. Wolf, Alan G. Palestine, Amit K. Reddy

**Affiliations:** 1 Department of Ophthalmology, University of Colorado School of Medicine, 1675 Aurora Court, F731, Aurora, CO 80045 USA; 2 Rocky Mountain Uveitis, Denver, CO USA; 3 Department of Neurology, University of Colorado School of Medicine, Aurora, CO USA

**Keywords:** Ofatumumab, Kesimpta, Multiple sclerosis, Uveitis, Retinal vasculitis

## Abstract

**Purpose:**

To report three cases of patients with multiple sclerosis (MS)-associated uveitis whose ocular inflammation responded well to ofatumumab.

**Case presentations:**

Case 1: A 26-year-old male with bilateral MS-associated occlusive retinal vasculitis presented with 1 + vitreous cell in both eyes and diffuse vascular leakage in both eyes on fluorescein angiography (FA). After treatment with ofatumumab for 19 months, there was significant improvement in vitreous cell and vascular leakage bilaterally. Case 2: A 41-year-old male with bilateral MS-associated intermediate uveitis presented with 2 + vitreous cell bilaterally. FA demonstrated bilateral vascular leakage with cystoid macular edema. After transitioning to ofatumumab from ocrelizumab and corticosteroid injections, exam remained quiet with resolved FA leakage bilaterally without further injections for 10 months. Case 3: A 51 year old female with MS initially presented with 2 + AC cell in the left eye and was started on mycophenolate mofetil with improvement but was unable to wean off topical corticosteroids. After switching to ofatumumab, she remained quiet on prednisolone acetate once daily in both eyes for nine months.

**Conclusion:**

While the benefits of ofatumumab have been well reported in MS, this is the first case report to describe the successful treatment of MS-associated uveitis with ofatumumab. Ofatumumab may prove to be an effective therapeutic option for these patients, with the additional benefit of self-administration. Further studies would be beneficial to identify the patients who would most benefit from this treatment.

## Background

Uveitis is a major cause of visual impairment and particularly impacts the working age population, accounting for nearly 10–15% of legal blindness in the United States [1, 2]. Its prevalence is higher in patients with multiple sclerosis (MS) compared to the general population [1, 3]. Various forms of uveitis have been associated with MS, including intermediate uveitis and retinal vasculitis [3]. The treatment of MS-associated uveitis typically involves collaborative efforts between the treating neurologist and ophthalmologist. Ideally, immunomodulatory therapy can be utilized that treats both the central nervous system (CNS) disease and the ocular inflammation, however this is not always possible. For example, tumor necrosis factor (TNF)-alpha inhibitors are efficacious in the treatment of uveitis but are contraindicated in patients with MS due to the risk for worsening demyelination [4, 5]. However, other biologic agents, such as anti-CD20 medications rituximab and ocrelizumab, can be effective for both diseases [6, 7]. 

Ofatumumab (Kesimpta; Novartis AG, Basel, Switzerland) is a novel anti-CD20 monoclonal antibody that was approved in 2020 for the treatment of relapsing forms of MS. Unlike rituximab and ocrelizumab, which are given via intravenous infusions, ofatumumab is self-administered via a sub-cutaneous monthly injection. Ofatumumab binds strongly to a CD20 membrane epitope that is distinct from rituximab and ocrelizumab [8]. The seminal MIRROR study assessing efficacy and safety outcomes of ofatumumab demonstrated an approximate 65% reduction in the cumulative number of new MS lesions for all ofatumumab dose groups compared to placebo [9]. Ofatumumab achieved near complete B-cell depletion at a lower concentration than any of the prior anti-CD20 monoclonal antibodies due to its higher potency and affinity to B cells [8]. 

Though its benefits in MS have been well studied, the effects of ofatumumab on intraocular inflammation have not yet been reported. This case series reports three novel cases of patients with MS-associated uveitis whose intraocular inflammation responded well to ofatumumab.

## Methods

A retrospective chart review was performed for patients with uveitis who also had a diagnosis of multiple sclerosis and were being treated with ofatumumab. They were transitioned to ofatumumab to specifically treat their multiple sclerosis. The study received approval from the Colorado Multiple Institutional Review Board and all research conformed to the tenets of the Declaration of Helsinki. Clinical data included best corrected visual acuity (BCVA), intraocular pressure (IOP), exam findings, and ocular imaging from initial presentation and final follow up visit, including degree of anterior chamber (AC) and vitreous inflammation graded according to Standardization of Uveitis Nomenclature criteria [10].

## Case presentations

### Case 1

A 26-year-old transgender male on hormone replacement therapy with a history of migraines on sumatriptan had a history of episodes of painful paresthesias and bilateral occlusive retinal vasculitis noted at age 19. MRI brain showed multiple T2 lesions and lumbar puncture showed oligoclonal bands. He was diagnosed with relapsing MS per 2017 McDonald criteria and was started on monthly ofatumumab injections by his neurologist.

**Fig. 1:**
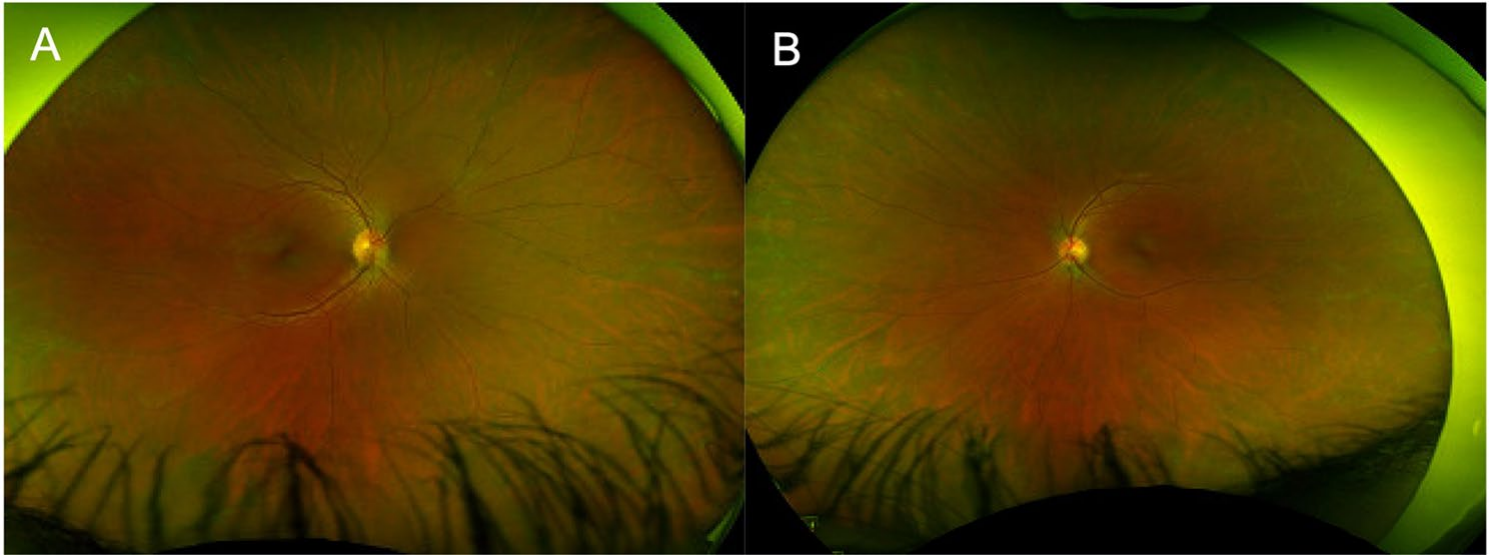
Fundus photos of right (**A**) and left (**B**) eye prior to ofatumumab initiation demonstrating vascular sheathing in both eyes with a hyperemic left optic disc

**Fig. 2:**
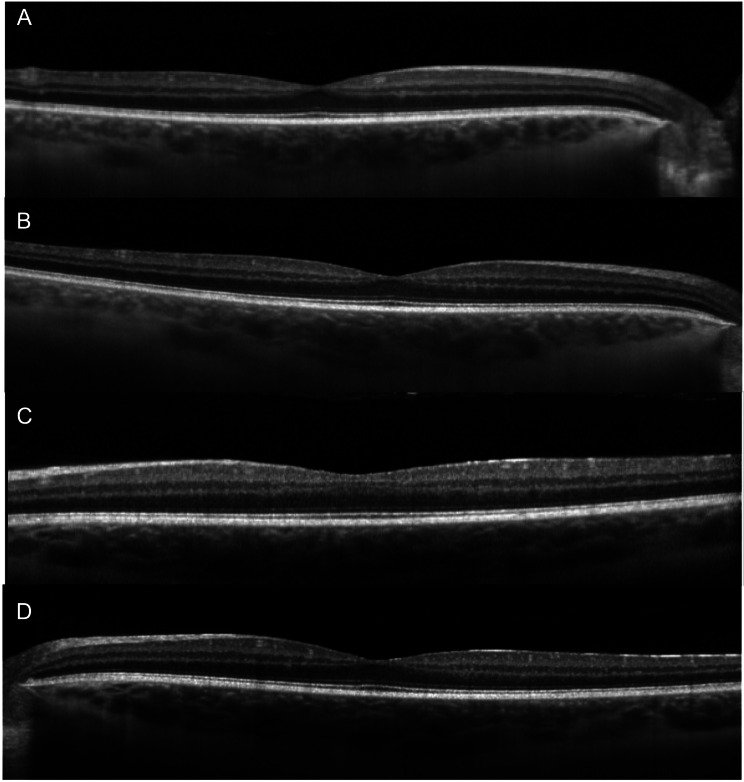
Optical coherence tomography of right (**A**) and left (**C**) eye prior to ofatumumab initiation demonstrating normal foveal contour without cystoid macular edema. 19 months after ofatumumab treatment, optical coherence tomography of right (**B**) and left (**D**) eye remained stable

**Fig. 3:**
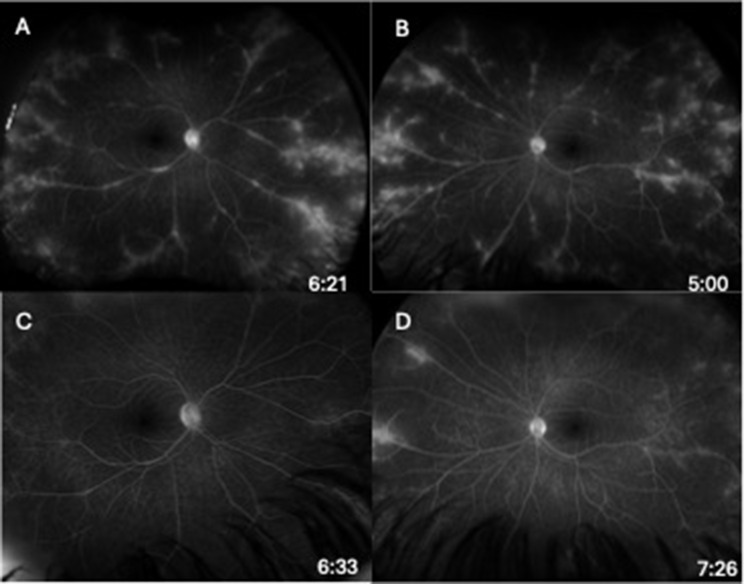
Late-phase fluorescein angiography of right (**A**) and left (**B**) eye prior to ofatumumab initiation demonstrating mild optic disc leakage, diffuse periphlebitis, and peripheral ferning with some peripheral areas of non-perfusion. Late-phase fluorescein angiography of right (**C**) and left (**D**) eye 19 months after ofatumumab treatment demonstrating significant improvement in optic disc leakage, diffuse periphlebitis and peripheral ferning

Initial ophthalmic evaluation five months prior to the start of ofatumumab showed visual acuities of 20/20 in both eyes with normal intraocular pressures. Exam was significant for a deep and quiet anterior chamber bilaterally, 1 + vitreous cells bilaterally and vascular sheathing in both eyes with a hyperemic left optic disc (Fig. 1A-B). Initial optical coherence tomography (OCT) demonstrated normal foveal contour with no cystoid macular edema (Fig. 2A and C). Initial FA showed mild optic disc leakage and diffuse periphlebitis and peripheral ferning in both eyes as well as areas of peripheral non-perfusion without neovascularization in both eyes (Fig. 3A-B).

Nineteen months after initiating ofatumumab, the patient’s MS was clinically and radiographically stable, and he denied any vision changes. His visual acuity remained 20/20 in both eyes with only trace vitreous cell in the right eye and rare vitreous cell in the left eye. His fundus exam showed vascular sheathing in both eyes and a hyperemic left optic disc. His OCT remained stable in both eyes (Fig. 2B and D). FA showed significant continued improvement in optic disc leakage, periphlebitis and peripheral ferning in both eyes (Fig. 3C-D).

### Case 2

A 41-year-old male initially presented with a long history of bilateral intermediate uveitis since the age of 19, complicated by cystoid macular edema (CME) in the left eye resulting in central macular atrophy. Previous treatments included topical and oral corticosteroids. His prior workup was negative for any systemic disease.

**Fig. 4:**
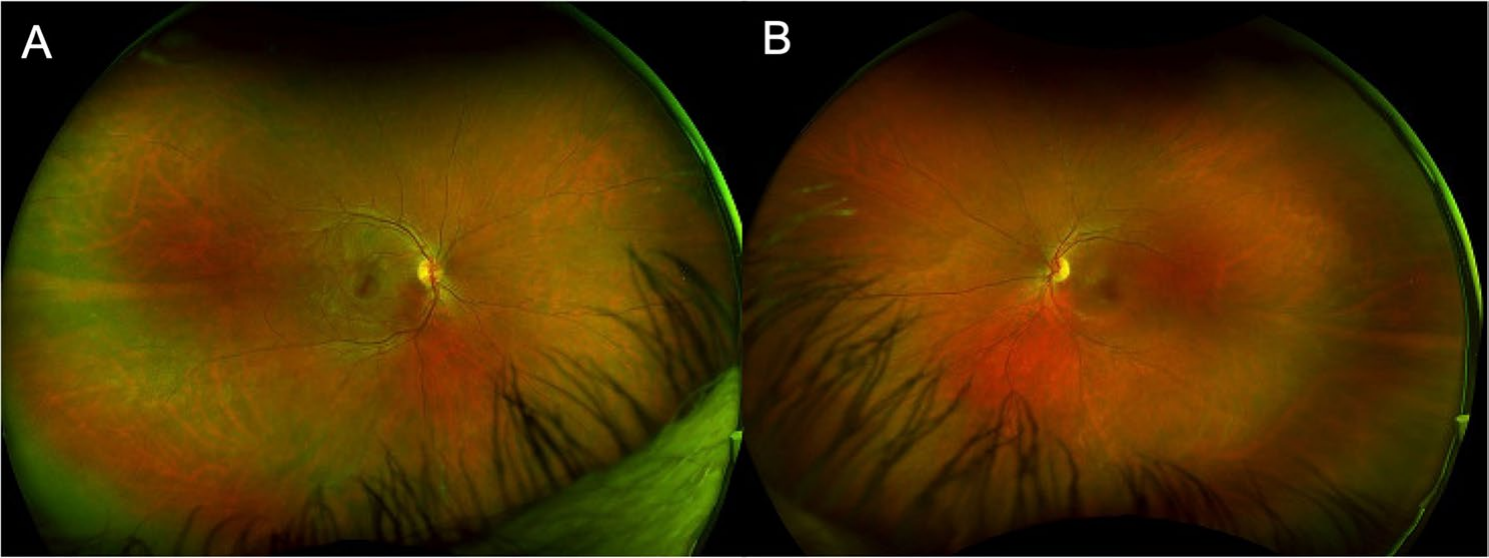
Fundus photos of right (**A**) and left (**B**) eye prior to ofatumumab initiation were within normal limits

**Fig. 5:**
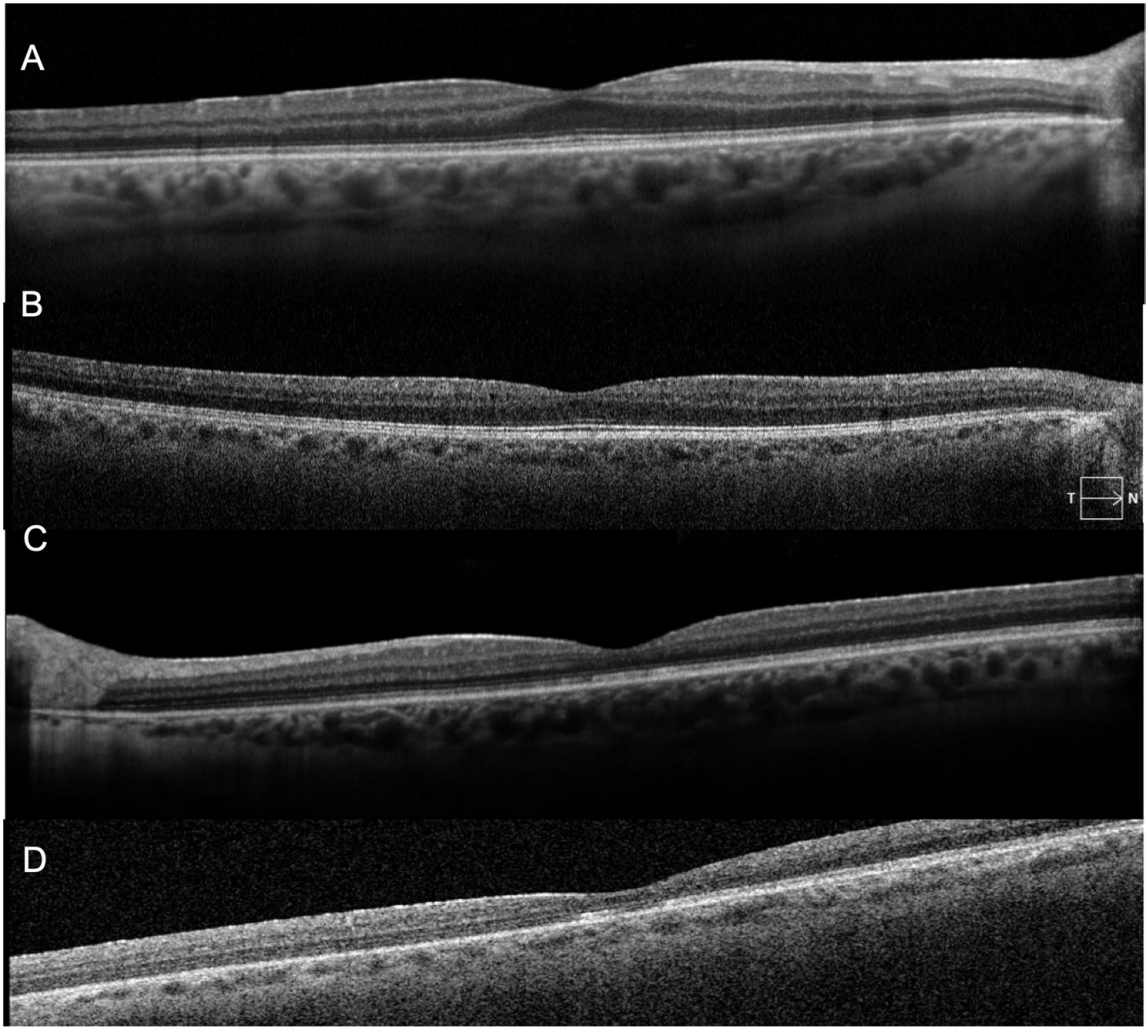
Optical coherence tomography of right (**A**) and left (**C**) eye prior to ofatumumab initiation were without cystoid macular edema, but demonstrated macular atrophy in the left eye. 10 months after ofatumumab treatment, optical coherence tomography of right (**B**) and left (**D**) eye remained stable

**Fig. 6:**
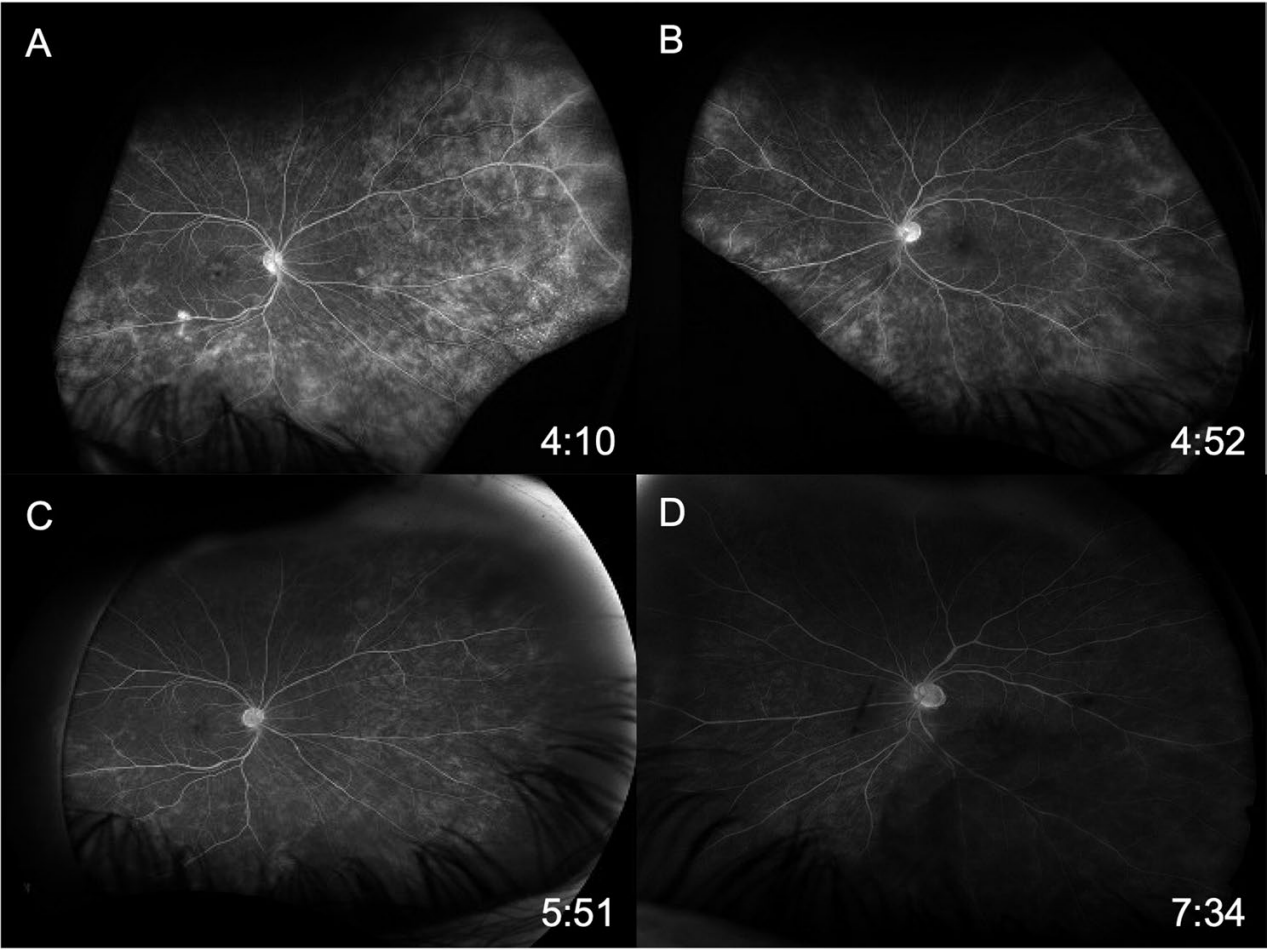
Late-phase fluorescein angiography of right (**A**) and left (**B**) eye prior to ofatumumab initiation demonstrating extensive bilateral vascular leakage with mild cystoid macular edema. Late-phase fluorescein angiography of right (**C**) and left (**D**) eye 10 months after ofatumumab treatment demonstrating resolution of the peripheral leakage on FA

Initial visual acuity was 20/20 in the right eye and 20/125 in the left eye with normal intraocular pressures bilaterally. Fundus exam showed bilateral 2 + anterior vitreous cell with moderate peripheral snowbanking. Color fundus photos were within normal limits (Fig. 4A-B). Initial optical coherence tomography showed no cystoid macular edema, but demonstrated macular atrophy in the left eye (Fig. 5A and C). Initial fluorescein angiogram (FA) demonstrated extensive bilateral vascular leakage with mild CME (Fig. 6A-B). Over the course of his follow-up, he received multiple local corticosteroid injections, including several bilateral sub-Tenon’s triamcinolone acetonide (STA) injections and bilateral 0.7-mg dexamethasone intravitreal implant injections, with improvement in vitreous cell and improved vascular leakage and CME on FA. He did not have an IOP rise in response to STA injections, but did have an IOP rise to the dexamethasone intravitreal implant injections requiring a short course of Dorzolamide-Timolol combination drop bilaterally twice per day with eventual normalization of intraocular pressures.

Three years after initial presentation, the patient developed balance issues, gait difficulty, episodes of facial and left arm numbness, and dysphagia. MRI brain showed active demyelinating plaques. He was formally diagnosed with multiple sclerosis per McDonald 2017 criteria and started on ocrelizumab 600 mg intravenous infusions every 6 months.

After the initiation of ocrelizumab, the patient still required ongoing yearly STA injections for persistent vascular leakage on FA, despite the MS being radiologically stable with slowed clinical progression. The patient was ultimately transitioned to monthly self-administered ofatumumab by his neurologist due to insurance barriers and difficulties with transportation to infusion appointments. During the 10 months following initiation of ofatumumab, the patient’s MS remained clinically and radiographically stable, and his uveitis remained quiet with resolved peripheral leakage on FA (Fig. 6C-D). His OCT remained stable in both eyes (Fig. 5B and D). He did not require further local corticosteroid injections during this time. Final visual acuity was 20/50 in the right eye and 20/200 in the left. A proposed theory for the decline in visual acuity in this patient may be multifactorial, including decades-long damage from previously insufficiently treated intraocular inflammation, refractive changes that likely require him to now wear spectacles, and epiretinal membrane changes in the right eye,

### Case 3

A 51 year old female, with a history of MS diagnosed at 41 years old and iridocyclitis diagnosed at 42 years old, initially presented with 2 + AC cell in the left eye and was started on prednisolone acetate 1% four times daily. She was subsequently started on methotrexate, which was discontinued due to gastrointestinal side effects. She was then transitioned to mycophenolate mofetil 1500 mg twice daily and was well controlled on prednisolone acetate 1% twice daily. However, multiple attempts at tapering prednisolone acetate led to recurrences of anterior uveitis. The patient’s neurologist started ofatumumab and requested cessation of mycophenolate. Three months after stopping mycophenolate and starting ofatumumab, she remained quiet on prednisolone acetate once daily. She continued to remain quiet on this regimen for a total of nine months after starting ofatumumab. Further attempts to decrease topical corticosteroids were not made at the request of her glaucoma provider, to assist in prevention of scarring around a prior tube shunt. Final visual acuity was 20/20 both eyes with normal intraocular pressures. The patient has remained under the care of the same uveitis specialist. While her clinical history, examinations and interpretation of prior imaging have remained in her medical record, the uveitis specialist moved practices and no longer had physical access to the patient images and diagnostic testing.

## Discussion

This case series described the clinical course of three patients with MS-associated uveitis whose intraocular inflammation responded well to ofatumumab monotherapy. Two patients with intermediate uveitis and retinal vasculitis achieved good control of vitreous cell and angiographic leakage, and the third patient with recurrent anterior uveitis maintained good control of AC cell on minimal topical corticosteroid therapy after initiating ofatumumab. This series is the first to demonstrate that ofatumumab may prove beneficial in the treatment of ocular inflammation in addition to CNS disease, and may reduce the need for adjunctive ocular corticosteroid therapy.

While the etiology of MS-associated uveitis has not been fully elucidated, various immune-mediated mechanisms have been postulated. Several theories have been proposed, including autoimmunity against myelin antigens, such as myelin basic protein (MBP), as well as non-myelin antigens co-expressed in the uvea and the CNS [1, 3, 11]. This can lead to a cross-reactivity that contributes to intraocular inflammation. Additionally, activated pathogenic T cells may fail to respond to regulatory cues, leading to the release of inflammatory mediators [1, 3, 11]. 

Newer evidence has shown that B cells play a crucial role in the pathogenesis of MS via antigen-driven autoantibody responses and through the cross regulation of T-helper cells. B cells have thus become a specific target for therapies to limit disease activity in patients with relapsing MS [12]. Various anti-CD20 antibody treatments for MS have been developed and proven successful, including rituximab, ublituximab, and ocrelizumab [6, 7, 12]. Compared to the alternative anti-CD20 therapies which are administered via infusions, ofatumumab offers a logistical advantage as a sub-cutaneous medication that can be administered at home. The seminal MIRROR study that assessed dose response effects of ofatumumab on safety and efficacy outcomes for MS demonstrated a 65% reduction in cumulative number of new lesions at week 12 [9]. Our reasoning is that radiographic stability of MS disease proven in this study with concurrent dose-dependent depletion of B cells would translate to minimal duration of therapy to control or treat MS-associated intraocular inflammation [9]. 

For MS-associated uveitis, short term treatments include corticosteroids – systemic, topical, periocular or intravitreal [11]. However, both local corticosteroid therapies are known to accelerate the development of cataracts and can increase the risk of glaucoma [13, 14]. Thus, steroid sparing immunosuppression is the preferred mode of treatment long term. However, TNF -alpha inhibitors such as adalimumab and infliximab are contraindicated in patients with MS, and the number of other immunosuppressive options that benefit both MS and uveitis are more limited. The knowledge that ofatumumab may be an effective treatment for both CNS and ocular disease will be beneficial to clinicians treating these complex patients.

This case series showed three promising examples of MS-associated uveitis that was well controlled on ofatumumab. Larger studies will be helpful to further explore the role of this medication in treating MS-associated uveitis.

## Conclusions

These cases highlight the potential role of ofatumumab in the treatment of MS-associated uveitis.

## Data Availability

The datasets used and/or analyzed during the current study are available from the corresponding author on reasonable request.

## Abbreviations

MS: Multiple Sclerosis
FA: Fluorescein Angiography
CNS: Central Nervous System
BCVA: Best corrected visual acuity
IOP: Intraocular pressure
AC: Anterior chamber
CME: Cystoid macular edema
STA: Sub-Tenon’s triamcinolone acetonide
MBP: Myelin basic protein
